# Supplementary material for: A Bayesian Informative Shrinkage Approach for Large-scale Multiple Hypothesis Testing (BISHOT): with Applications in Differential Analysis of Omics Data
Source: bioRxiv. 2025 Nov 29:2025.09.11.675690. Originally published 2025 Sep 16. Preprint. [Version 2] doi: 10.1101/2025.09.11.675690 (PMC12458450; doi:10.1101/2025.09.11.675690)
Supplement: Supplement 1 [file media-1.pdf]

Supplementary Material to *A Bayesian Informative Shrinkage Approach for Large-scale Multiple Hypothesis Testing (BISHOT): with Applications in Differential Analysis of Omics Data*

Ya Su and Mary Eunice Joy Z. Clark
Department of Statistical Sciences and Operational Research, Virginia Commonwealth University, Richmond, VA 23284-3083, U.S.A.
Chi Wang
Division of Cancer Biostatistics, Department of Internal Medicine and Markey Cancer Center, University of Kentucky, Lexington, 40536, U.S.A.

S.1 Appendix A

S.1.1 Proof of Theorem 1

We first calculate the expected value of $L_{1,\lambda}(\boldsymbol{\delta}, \boldsymbol{\omega})$ under the posterior distribution of $p(\boldsymbol{\omega}|D)$:

$$E_{\boldsymbol{\omega}|D}\{L_{1,\lambda}(\boldsymbol{\delta}, \boldsymbol{\omega})\} = \frac{1}{G}\sum_{g=1}^{G}\{\lambda\widetilde{\omega}_{0g}\delta_g + \widetilde{\omega}_{1g}(1-\delta_g)\}.$$

The optimal choices for δ_g would be $\delta_g = I\{\lambda\widetilde{\omega}_{0g} < \widetilde{\omega}_{1g}\}$.

Repeat the above process for $L_{2,\lambda}$, the optimal choices for δ_g corresponding to $L_{2,\lambda}$ is $\delta_g = I\{\lambda\widetilde{\omega}_{0g} < \widetilde{\omega}_{2g}\}$.

S.1.2 Proof of Theorem 2

The proof is similar to that of Theorem 1.

S.1.3 Proof of Theorem 3

The proof extends that of Theorem 1 in Fu et al. (2022) in the framework of the proposed Bayesian procedure. We first prove two properties about $\alpha(\lambda) = \mathrm{SFDR}(\boldsymbol{\delta}^{OPT})$: (i) $\alpha(\lambda) < \lambda$ and (ii) $\alpha(\lambda)$ is non-decreasing.

For any α given, suppose λ is chosen so that $\mathrm{mSFDR}(\boldsymbol{\delta}^{OPT}) = \alpha$. For an arbitrary decision $\boldsymbol{d} = (d_1, \ldots, d_G)$ satisfying $\mathrm{SFDR}(\boldsymbol{d}) \leq \alpha$. Equivalently,

$$\begin{aligned} E\bigg(\sum_{g\in\mathcal{S}}\{(1-\alpha)\widetilde{\omega}_{0g} - \alpha\widetilde{\omega}_{1g}\}\delta_g^{OPT} + \sum_{g\in\mathcal{S}^c}\{(1-\alpha)\widetilde{\omega}_{0g} - \alpha\widetilde{\omega}_{2g}\}\delta_g^{OPT}\bigg) &= 0 \\ E\bigg(\sum_{g\in\mathcal{S}}\{(1-\alpha)\widetilde{\omega}_{0g} - \alpha\widetilde{\omega}_{1g}\}d_g + \sum_{g\in\mathcal{S}^c}\{(1-\alpha)\widetilde{\omega}_{0g} - \alpha\widetilde{\omega}_{2g}\}d_g\bigg) &\leq 0. \end{aligned}$$

Immediately,

$$E\bigg(\sum_{g\in\mathcal{S}}\{(1-\alpha)\widetilde{\omega}_{0g} - \alpha\widetilde{\omega}_{1g}\}(\delta_g^{OPT} - d_g) + \sum_{g\in\mathcal{S}^c}\{(1-\alpha)\widetilde{\omega}_{0g} - \alpha\widetilde{\omega}_{2g}\}(\delta_g^{OPT} - d_g)\bigg) \geq 0. \quad \text{(S.1)}$$

Let $t = (\lambda - \alpha)/(1-\lambda) > 0$ (from (i)). If $g \in \mathcal{S}$, when $\delta_g^{OPT} = 1$, we have $\widetilde{\omega}_{0g} < \lambda(\widetilde{\omega}_{0g} + \widetilde{\omega}_{1g})$, equivalently, $t\widetilde{\omega}_{1g} > (1-\alpha)\widetilde{\omega}_{0g} - \alpha\widetilde{\omega}_{1g}$, otherwise when $\delta_g^{OPT} = 0$, $t\widetilde{\omega}_{1g} \leq (1-\alpha)\widetilde{\omega}_{0g} - \alpha\widetilde{\omega}_{1g}$. Hence $\sum_{g\in\mathcal{S}}\{(1-\alpha)\widetilde{\omega}_{0g} - \alpha\widetilde{\omega}_{1g}\}(\delta_g^{OPT} - d_g) \leq t\sum_{g\in\mathcal{S}}\widetilde{\omega}_{1g}(\delta_g^{OPT} - d_g)$. Similarly, $\sum_{g\in\mathcal{S}^c}\{(1-\alpha)\widetilde{\omega}_{0g} - \alpha\widetilde{\omega}_{2g}\}(\delta_g^{OPT} - d_g) \leq t\sum_{g\in\mathcal{S}^c}\widetilde{\omega}_{2g}(\delta_g^{OPT} - d_g)$. Together with (S.1),

$$E\bigg(\sum_{g\in\mathcal{S}}\widetilde{\omega}_{1g}(\delta_g^{OPT} - d_g) + \sum_{g\in\mathcal{S}^c}\widetilde{\omega}_{2g}(\delta_g^{OPT} - d_g)\bigg) \geq 0.$$

This indicates that $\mathrm{SETP}(\boldsymbol{\delta}^{OPT}) \geq \mathrm{SETP}(\boldsymbol{d})$ and concludes the proof.

S.1.4 Connection of BCR to Bayes factor

Recall $\Theta_0 = (-\epsilon, \epsilon)$, $\Theta_1 = (-\infty, -\epsilon)$ and $\Theta_2 = (\epsilon, \infty)$. Let $\Pi|_{\Theta_i}(\cdot) = \Pi(\cdot)/\Pi(\Theta_i)$ be the prior distribution Π restricted on Θ_i $(i = 0, 1, 2)$, and the marginal distribution of data under H_i is $\Pr(D|H_i) = \int_{\beta\in\Theta_i}\Pi|_{\Theta_i}(\beta)P(D|\beta)d\beta$ $(i = 0, 1, 2)$, therefore

$$\mathrm{BF}_{i0} = \frac{\Pr(D|H_i)}{\Pr(D|H_0)} = \frac{\int_{\beta\in\Theta_i}\Pi(\beta)\Pr(D|\beta)d\beta}{\int_{\beta\in\Theta_0}\Pi(\beta)\Pr(D|\beta)d\beta}\frac{\Pi(\Theta_0)}{\Pi(\Theta_i)} \text{ for } i = 1, 2.$$

Without loss of generality, let us assume that $\Pi(\Theta_1) = \Pi(\Theta_2)$. From the expression of BCR in Section 2.2, we immediately find that $C_\beta = 1/(1 + \Pi(\Theta_1) \cdot \Pi(\Theta_0)^{-1} \cdot \max\{\mathrm{BF}_{10}, \mathrm{BF}_{20}\})$.

S.1.5 Conditional distributions of parameters in models (1) and (3)

$$\Pr(c_g, \beta_g | \lambda_g, \tau, D_g) \sim \mathrm{t}_{\nu_g}(\mu_g, s_g)$$
$$\Pr(\lambda_g \,|\beta_g, \tau, \sigma_g^2, h_g) \propto \frac{1}{\lambda_g(1+\lambda_g^2)} \exp\left\{ -\frac{(\beta_g - h_g)^2}{2\lambda_g^2\tau^2\sigma_g^2} \right\} I(\lambda_g > 0)$$
$$\Pr(\tau \,|\beta_1, \dots, \beta_G, \sigma_1^2, \dots, \sigma_G^2, h_1, \dots, h_G) \propto \frac{1}{(1+\tau^2)\tau^G} \exp\left\{ -\sum_{g=1}^{G} \frac{(\beta_g - h_g)^2}{2\lambda_g^2\tau^2\sigma_g^2} \right\} I(\tau > 0)$$
$$\Pr(\sigma_g^2 | \beta_g, \lambda_g, \tau, h_g) \sim \mathrm{IG}(\widetilde{\xi}_1, \widetilde{\xi}_2),$$

where $\mu_g = Q_g^{-1}\eta_g$ with $Q_g = \sum_i \widetilde{X}_i \widetilde{X}_i^\top + \mathrm{diag}(100^{-1}, \lambda_g^{-2}\tau^{-2})$ and $\eta_g = \sum_i \widetilde{X}_i Y_{ig} + \lambda_g^{-2}\tau^{-2}h_g$, $s_g = Q_g^{-1}\{\xi_1 + n/2\}^{-1}\{\xi_2 + (\sum_i Y_{ig}^2 + \lambda_g^{-2}\tau^{-2}h_g^2 - \eta_g^\top Q_g^{-1}\eta_g)/2\}$ and $\nu_g = 2\xi_1 + n$. $\mathrm{t}_\nu(\mu, s)$ denotes a multivariate t distribution with center μ and scale s and degrees of freedom ν, $\widetilde{\xi}_1 = \xi_1 + n/2 + 1$, $\widetilde{\xi}_2 = \xi_2 + (c_g^2/10^2 + (\beta_g - h_g)^2\tau^{-2}\lambda_g^{-2} + \sum_i (Y_{ig} - c_g - X_i^\top \beta_g)^2)/2$. Here $\widetilde{X}_i$ is $(1, X_i^\top)^\top$ which expands X_i with a constant 1.

S.1.6 Simulation II and results

In simulation II, we adopt the same mechanism for the set of DE genes Ω but try to explore a scenario when the DE gene differential expressions has a unimodal distribution at zero, which indicates that true DE expressions could be close to zero rather than a distance away from zero. Specifically, if $g \in \Omega$, $\beta_g \sim 0.4\mathrm{N}(0, 0.25^2) + 0.2\mathrm{N}(0, 0.5^2) + 0.2\mathrm{N}(0, 1^2) + 0.2\mathrm{N}(0, 2^2)$. This DE distribution is the same as the spiky scenario in Stephens (2017).

The parameters needed in model (1) are set as follows: $c_g = 4$, $\sigma_g = 1.5$ for a total $G = 5000$ genes. ϵ is set to be $\epsilon = 0.01$ or $\epsilon = 0.05$ and $h_g \sim \mathrm{N}(\beta_g, 0.1^2)$ in reflection of the size of the true DE genes generated above (ϵ and h_g should be set to be adaptive to the distribution of true DE genes practically). The

remaining parameter/hyperparameter choices are identical to the description in Section 4. See Figures S.1-S.4 for the similar analyses comparing BISHOT and SunSpike0 as presented in Section 4 based on 50 simulated data sets.

As observed in Section 4, BISHOT is more robust among all scenarios spanned by p and n in producing SFDR estimates and accuracy in top ranked genes. BISHOT appears to produce some "fake" discoveries when ϵ is larger and the SFDR nominal threshold is small (right panel in Figure S.2). After close examination these are actual nonzero β_g that are smaller than but close to ϵ, whose identification are caused by their priors sticking out. This shall suggest that we select ϵ appropriately by not neglecting the bulk of the distribution of β_g, e.g. the middle probability corresponding to $|\beta_g| < \epsilon(= 0.05)$ is about 10% which, when combined with a relative large prior h_g, can possibly lead to the aforementioned "fake" discoveries.

S.2 Appendix B

S.2.1 Venn diagram comparing DE results from BISHOT and SunSpike0 in Section 5

S.2.2 Enriched GO terms from the DE analysis in Section 6

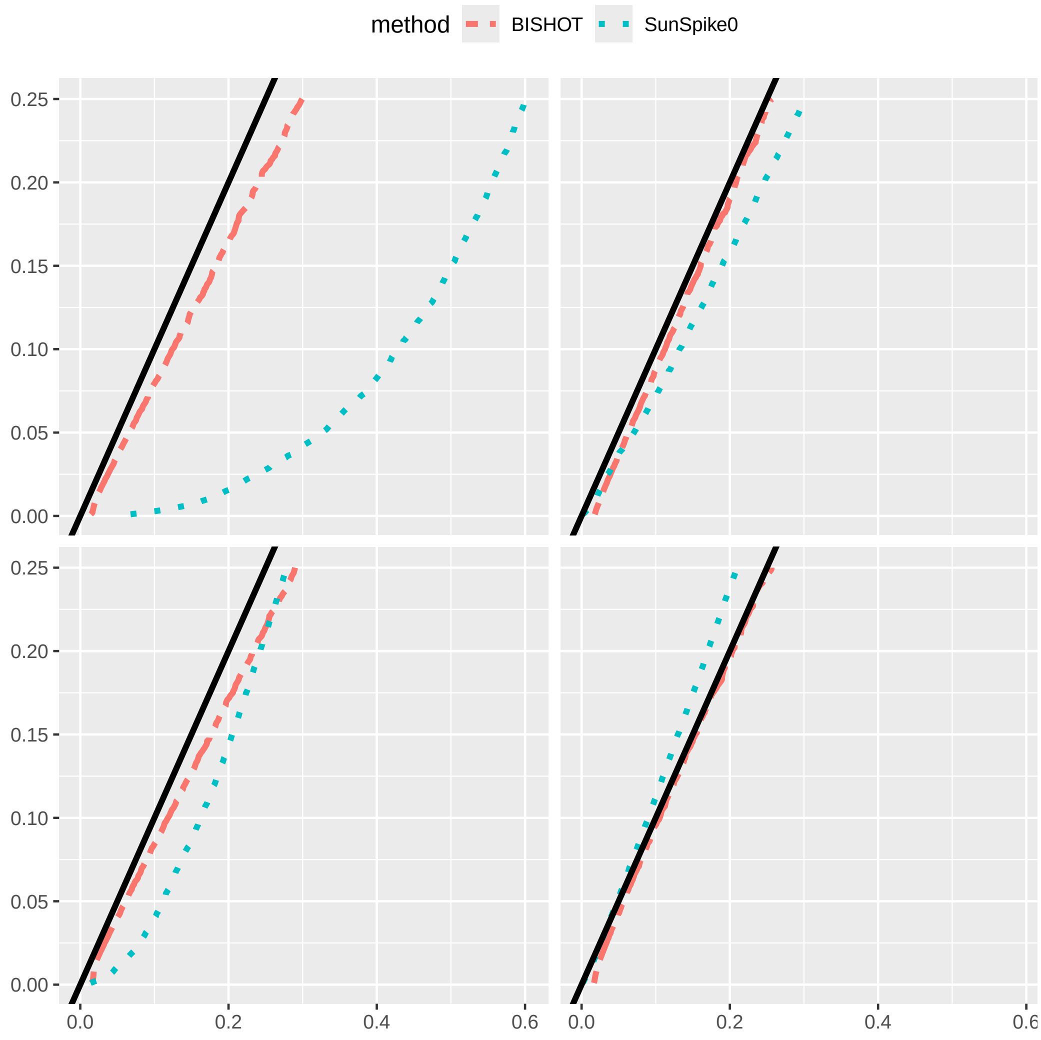

Figure S.1: The actual signed false discovery rate (SFDR) when $\epsilon = 0.01$ averaged over 50 simulated data using mSFDR threshold 0.001 to 0.25 corresponding to 10 (left panel) or 50 (right panel) samples in the treatment and control group. The top and bottom panel displays the case when $p = 0.1$ and $p = 0.5$.

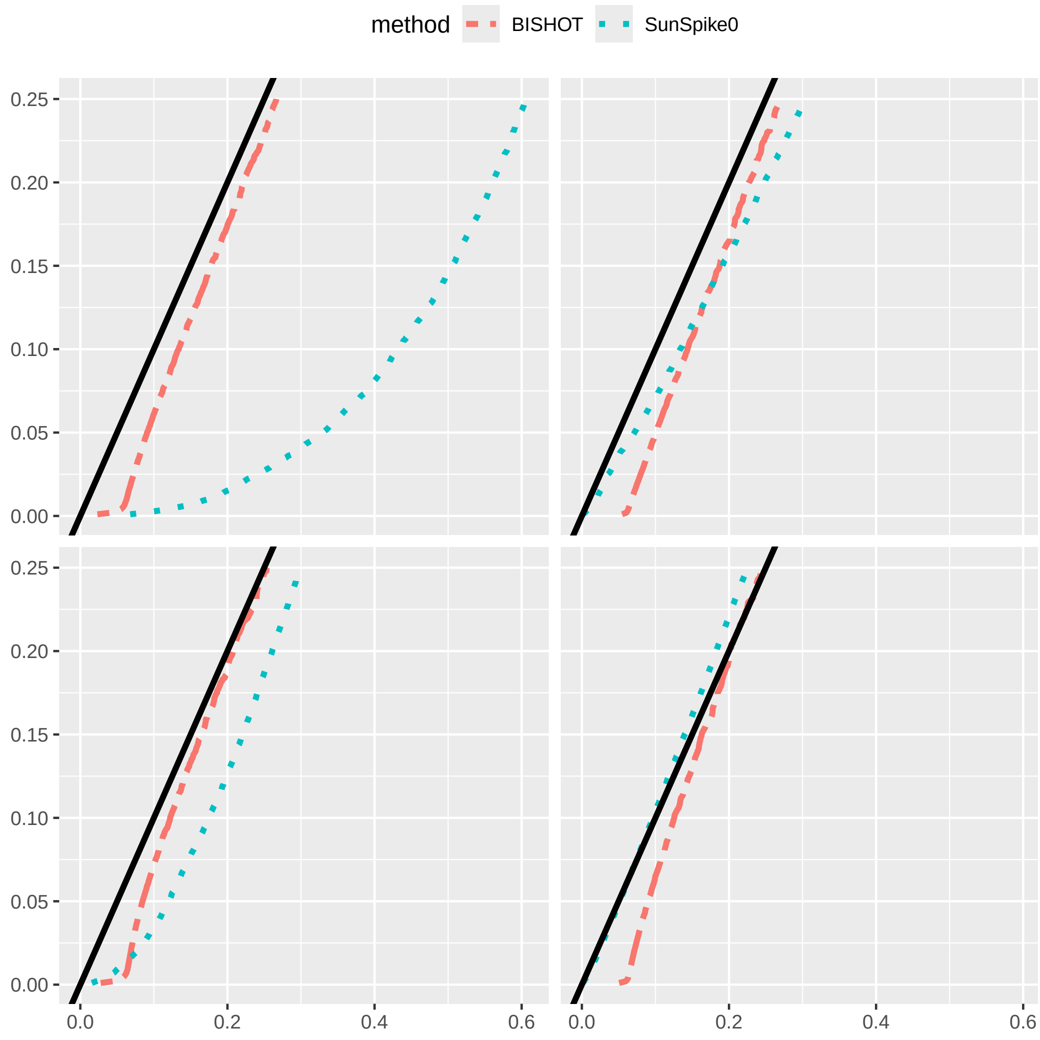

Figure S.2: The actual signed false discovery rate (SFDR) when $\epsilon = 0.05$ averaged over 50 simulated data using mSFDR threshold 0.001 to 0.25 corresponding to 10 (left panel) or 50 (right panel) samples in the treatment and control group. The top and bottom panel displays the case when $p = 0.1$ and $p = 0.5$.

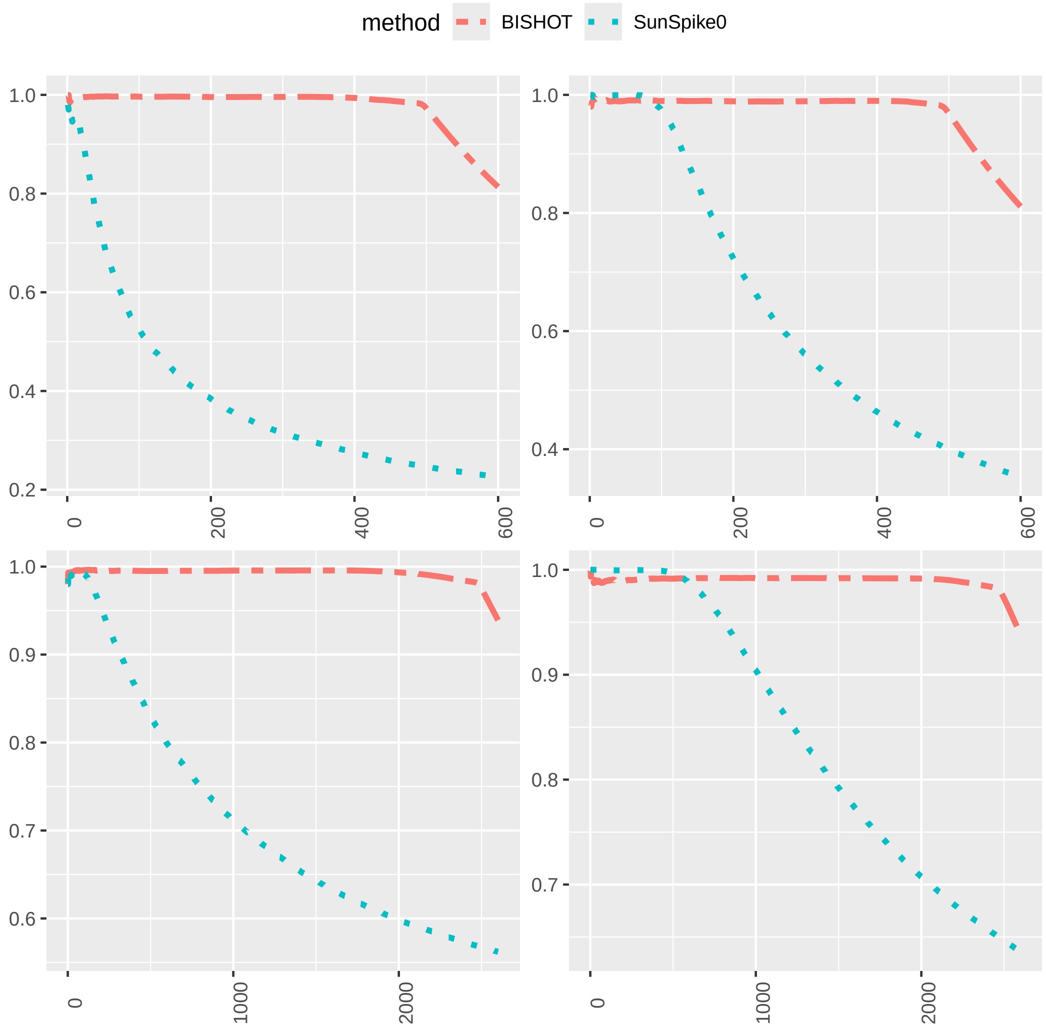

Figure S.3: The proportion of true DE genes for the first R genes with smallest q values when $\epsilon = 0.01$ averaged over 50 simulated data for $R = 1, \ldots, 5000p+100$ corresponding to 10 (left panel) or 50 (right panel) samples in the treatment and control group. The top and bottom panel displays the case when $p = 0.1$ and $p = 0.5$.

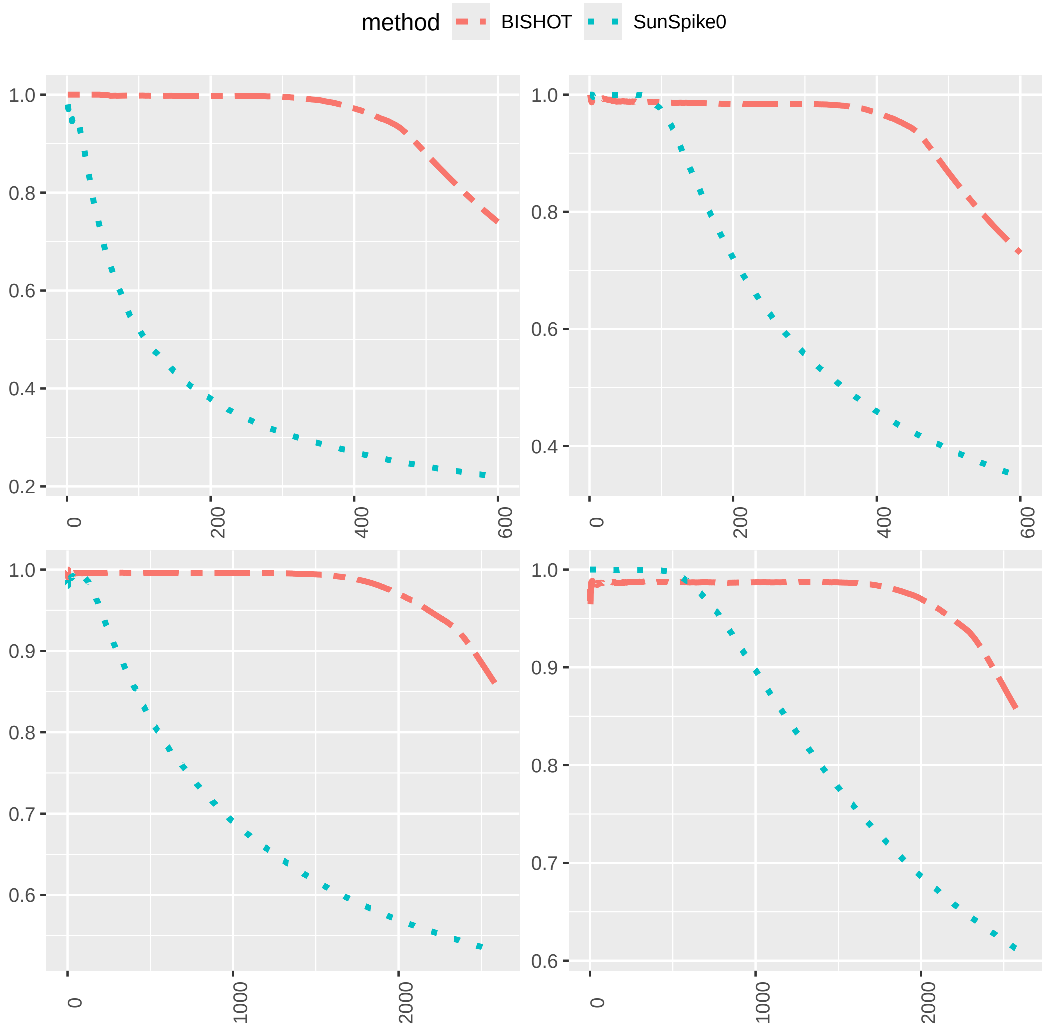

Figure S.4: The proportion of true DE genes for the first R genes with smallest q values when $\epsilon = 0.05$ averaged over 50 simulated data for $R = 1, \ldots, 5000p+100$ corresponding to 10 (left panel) or 50 (right panel) samples in the treatment and control group. The top and bottom panel displays the case when $p = 0.1$ and $p = 0.5$.

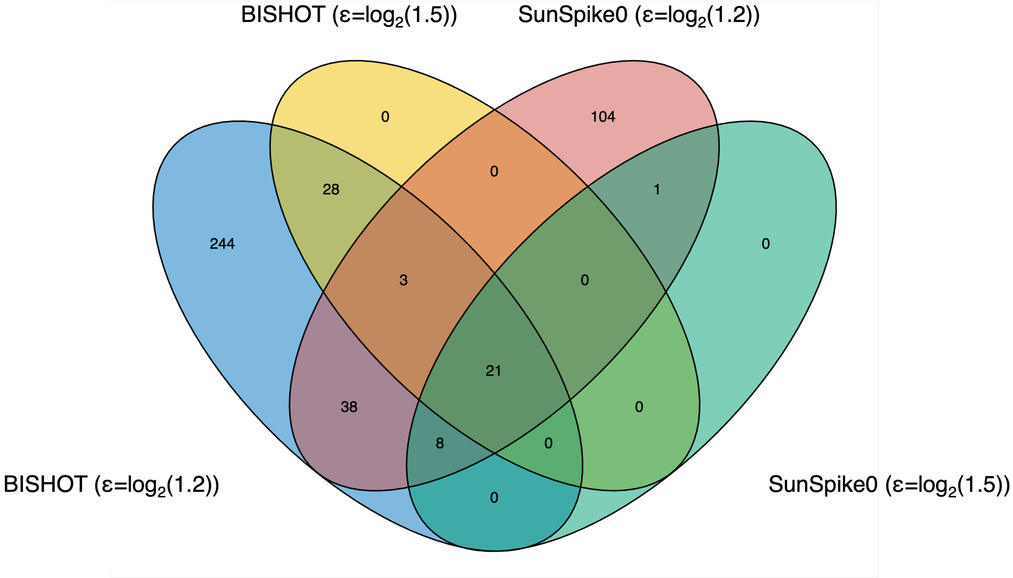

Figure S.5: Venn diagram comparing DE genes identified by different methods from the DE analysis in Section 5.

GO Term ID	Term Name	Category	Alteration
GO:0000902	cell morphogenesis	Biological Process	up-regulated
GO:0004674	protein serine/threonine kinase activity	Molecular Function	up-regulated
GO:0030010	establishment of cell polarity	Biological Process	up-regulated
GO:0004672	protein kinase activity	Molecular Function	up-regulated
GO:0002399	spleen development	Biological Process	up-regulated
GO:0045669	positive regulation of cell differentiation	Biological Process	up-regulated
GO:0048589	developmental growth	Biological Process	up-regulated
GO:0004674	protein serine/threonine kinase activity	Molecular Function	up-regulated
GO:0008361	regulation of cell size	Biological Process	up-regulated
GO:0048638	regulation of developmental growth	Biological Process	up-regulated
GO:0061028	establishment of endothelial barrier	Biological Process	up-regulated
GO:0010494	cytoplasmic stress granule	Cellular Component	up-regulated
GO:0007017	microtubule-based process	Biological Process	up-regulated
GO:0045071	negative regulation of viral genome replication	Biological Process	up-regulated
GO:0003730	mRNA 3'-UTR binding	Molecular Function	up-regulated
GO:0060560	developmental growth involved in morphogenesis	Biological Process	up-regulated
GO:0009790	embryo development	Biological Process	up-regulated
GO:0003682	chromatin binding	Molecular Function	up-regulated
GO:0009887	animal organ morphogenesis	Biological Process	up-regulated
GO:0004812	aminoacyl-tRNA ligase activity (aminoacyltransferase activity)	Molecular Function	up-regulated
GO:0048471	perinuclear region of cytoplasm	Cellular Component	up-regulated
GO:0048588	developmental cell growth	Biological Process	up-regulated
GO:0008610	lipid biosynthetic process	Biological Process	up-regulated
GO:0001223	transcription coactivator binding	Molecular Function	up-regulated
GO:0097542	ciliary tip	Cellular Component	up-regulated
GO:0040007	growth	Biological Process	up-regulated
GO:0044782	cilium organization	Biological Process	up-regulated
GO:0060041	retina morphogenesis in camera-type eye	Biological Process	up-regulated
GO:0009792	embryo development ending in birth or egg hatching	Biological Process	up-regulated
GO:0001570	vasculogenesis	Biological Process	up-regulated
GO:0051020	GTPase binding	Molecular Function	up-regulated
GO:0006412	translation	Biological Process	down-regulated
GO:0005762	mitochondrial large ribosomal subunit	Cellular Component	down-regulated
GO:0022613	ribonucleoprotein complex biogenesis	Biological Process	down-regulated
GO:0140053	mitochondrial gene expression	Biological Process	down-regulated
GO:0002181	cytoplasmic translation	Biological Process	down-regulated
GO:0016469	proton-transporting two-sector ATPase complex	Cellular Component	down-regulated
GO:0042254	ribosome biogenesis	Biological Process	down-regulated
GO:0050819	regulation of coagulation	Biological Process	down-regulated
GO:0005791	rough endoplasmic reticulum	Cellular Component	down-regulated
GO:0005761	mitochondrial small ribosomal subunit	Cellular Component	down-regulated
GO:1990908	endonuclease complex	Cellular Component	down-regulated
GO:0061077	chaperone-mediated protein folding	Biological Process	down-regulated
GO:0009141	nucleoside triphosphate metabolic process	Biological Process	down-regulated
GO:0042274	ribosomal small subunit biogenesis	Biological Process	down-regulated
GO:0006457	protein folding	Biological Process	down-regulated
GO:0000955	RNA 5'-end processing	Biological Process	down-regulated
GO:0005771	multivesicular body	Cellular Component	down-regulated
GO:0006364	rRNA processing	Biological Process	down-regulated
GO:0032040	small-subunit processome	Cellular Component	down-regulated
GO:0031970	organelle envelope lumen	Cellular Component	down-regulated
GO:0051082	unfolded protein binding	Molecular Function	down-regulated
GO:0016072	rRNA metabolic process	Biological Process	down-regulated
GO:0044183	protein folding chaperone	Molecular Function	down-regulated
GO:0007006	mitochondrial membrane organization	Biological Process	down-regulated
GO:0051087	protein folding chaperone binding	Molecular Function	down-regulated
GO:0032509	endosome transport via multivesicular body sorting pathway	Biological Process	down-regulated
GO:0097525	spliceosomal tri-snRNP complex	Cellular Component	down-regulated
GO:0071985	multivesicular body sorting pathway	Biological Process	down-regulated
GO:0006397	mRNA processing	Biological Process	down-regulated

Figure S.6: A list of enriched GO terms identified by BISHOT from the DE analysis in Section 6.
